# Polymicrobial Nature of Tick-Borne Diseases

**DOI:** 10.1128/mBio.02055-19

**Published:** 2019-09-10

**Authors:** Santiago Sanchez-Vicente, Teresa Tagliafierro, James L. Coleman, Jorge L. Benach, Rafal Tokarz

**Affiliations:** a Department of Molecular Genetics and Microbiology and Center for Infectious Diseases, Stony Brook University, Stony Brook, New York, USA; b Center for Infection and Immunity, Mailman School of Public Health, Columbia University, New York, New York, USA; Albert Einstein College of Medicine; University of Maryland School of Medicine; Yale School of Medicine; University of Minnesota; Tufts University School of Veterinary Medicine

**Keywords:** *Amblyomma*, *Anaplasma*, *Babesia*, *Borrelia burgdorferi*, *Ehrlichia*, *Ixodes*, Lyme disease, Powassan, *Rickettsia*

## Abstract

Tick-borne diseases have doubled in the last 12 years, and their geographic distribution has spread as well. The clinical spectrum of tick-borne diseases can range from asymptomatic to fatal infections, with a disproportionate incidence in children and the elderly. In the last few years, new agents have been discovered, and genetic changes have helped in the spread of pathogens and ticks. Polymicrobial infections, mostly in Ixodes scapularis, can complicate diagnostics and augment disease severity. Amblyomma americanum ticks have expanded their range, resulting in a dynamic and complex situation, possibly fueled by climate change. To document these changes, using molecular biology strategies for pathogen detection, an assessment of 12 microbes (9 pathogens and 3 symbionts) in three species of ticks was done in Suffolk County, New York. At least one agent was detected in 63% of I. scapularis ticks. Borrelia burgdorferi was the most prevalent pathogen (57% in adults; 27% in nymphs), followed by Babesia microti (14% in adults; 15% in nymphs), Anaplasma phagocytophilum (14% in adults; 2% in nymphs), Borrelia miyamotoi (3% in adults), and Powassan virus (2% in adults). Polymicrobial infections were detected in 22% of I. scapularis ticks, with coinfections of B. burgdorferi and B. microti (9%) and of B. burgdorferi and A. phagocytophilum (7%). Three *Ehrlichia* species were detected in 4% of *A. americanum* ticks. The rickettsiae constituted the largest prokaryotic biomass of all the ticks tested and included Rickettsia amblyommatis, Rickettsia buchneri, and Rickettsia montanensis. The high rates of polymicrobial infection in ticks present an opportunity to study the biological interrelationships of pathogens and their vectors.

## Abstract (precis)

Tick-borne diseases have increased in prevalence in the United States and abroad. The reasons for these increases are multifactorial, but climate change is likely to be a major factor. One of the main features of the increase is the geographic expansion of tick vectors, notably *Amblyomma americanum*, which has brought new pathogens to new areas. The clinical spectrum of tick-borne diseases can range from asymptomatic to fatal infections, with a disproportionate incidence in children and the elderly. In addition, new pathogens that are cotransmitted by Ixodes scapularis have been discovered and have led to difficult diagnoses and to disease severity. Of these, Borrelia burgdorferi, the agent of Lyme disease, continues to be the most frequently transmitted pathogen. However, Babesia microti, Borrelia miyamotoi (another spirochete), Anaplasma phagocytophilum, and Powassan virus are frequent cotransmitted agents. Polymicrobial infection has important consequences for the diagnosis and management of tick-borne diseases.

## INTRODUCTION

Polymicrobial infections are known for a variety of disorders, such as urinary tract infections, sexually transmitted diseases, periodontal disease, otitis media, and opportunistic infections associated with hospital procedures, to name just a few. In these settings, coexisting microbes can generate synergy or interference of the infectious process. However, regardless of the outcome of a specific polymicrobial infection, the interrelationships among the microbes are likely to have an impact on the course of the infection. Unlike some of the examples above where polymicrobial infections affect only the patient, the various pathogens and symbionts of ticks interact in both the tick itself and in the patient with consequences that have not been totally foreseen (1).

Tick-borne diseases have become a worldwide threat to public health. In the United States, cases increased from 22,000 in 2004 to >48,000 in 2016 (2). Tick-borne diseases range from subclinical to fatal infections, with a disproportionate incidence in children and the elderly. Moreover, some can also be transmitted by blood transfusions and cause severe disease in patients with underlying disorders. New agents have also been discovered (3, 4). Notably, formerly geographically confined tick species have expanded their range, resulting in a dynamic and complex situation, possibly fueled by climate change. Polymicrobial infections represent another aspect of tick-borne diseases that can complicate diagnosis and augment disease severity. Since the discovery of Borrelia burgdorferi in 1982 (5) and human babesiosis caused by the hemoprotozoan Babesia microti (6, 7) in Long Island and Nantucket in the late 1970s, more cotransmitted infections have been recognized. Two early serosurveys disclosed a link between babesiosis and erythema migrans and double infections (8, 9). Since then, Ixodes scapularis has been implicated as the vector of five human pathogens in the northeast United States. Polymicrobial infections occur in both North America and Europe (10).

Over the past 3 decades, there has been a steady increase in the number of newly discovered tick-borne agents. In addition to B. burgdorferi and B. microti, Anaplasma phagocytophilum (11–15), which was originally classified as a granulocytic *Ehrlichia* species (16), a relapsing fever-like *Borrelia* species, Borrelia miyamotoi (3), and the deer tick virus, a variant of Powassan virus (POWV) (4), are also transmitted by I. scapularis. POWV was first isolated from a patient with encephalitis (17), and over 100 cases have been reported in the United States (18). The capacity of I. scapularis to harbor such a diverse pathogen microbiome increases the risk of polymicrobial infections from a single tick bite. Almost 25 years ago, Telford et al. warned that cotransmission of pathogens will have a unique impact on public health in sites of endemicity (19).

Pathogens transmitted by Amblyomma americanum and Dermacentor variabilis contribute to the broad spectrum of tick-borne diseases. *A. americanum*, the lone star tick, has historically been found in the southern United States, but it has substantially expanded its range. Anecdotally, it appears that this aggressive species has become the most abundant tick in Long Island, New York, and in other regions previously outside its range (20, 21), and it has done so in a very short time (22, 23). A collateral effect of the expansion of *A. americanum* could be the displacement of I. scapularis and *D. variabilis* through competitive interactions that are not understood. Significant shifts in disease prevalence in the future could be due to shifts in the vector populations, and systematic tick-pathogen surveys may answer this question. *A. americanum* is a vector of Ehrlichia chaffeensis and Ehrlichia ewingii (24–27), both capable of causing severe disease in patients who are elderly or immunodeficient (28). *A. americanum* has been linked to a Lyme disease-like syndrome called southern tick-associated rash illness (STARI). Borrelia lonestari, a relapsing fever-like species found in *A. americanum* (29), has been associated with STARI, but its role has not been corroborated. Most recently, *A. americanum* has been implicated in meat allergy syndrome, an intriguing condition that may be due to this tick directly without the intervention of a microbe (30). Haemaphysalis longicornis, a newly discovered exotic tick species introduced in the United States (31), is an important vector of human and animal disease agents in its original geographic range (32, 33).

Of note is the increasing awareness of the rickettsial biome in the three vector ticks. The American dog tick, *D. variabilis,* is a vector of Rickettsia rickettsii, the agent of Rocky Mountain spotted fever (RMSF). The number of reported cases of spotted fever rickettsioses (SFR) in the United States has increased from 1,713 in 2004 to 4,269 in 2016 (2). However, tick surveillance studies have rarely reported *R. rickettsii* in *D. variabilis* (34, 35). This suggests that other species of *Rickettsia* contribute to the rise in incidence of SFR.

Rickettsia buchneri, an ovarian symbiont, (36), is the most abundant prokaryote in I. scapularis (37–39). Rickettsia amblyommatis (40) is pervasively associated with *A. americanum* (41). Rickettsia montanensis in *D. variabilis* has been implicated circumstantially in an RMSF-like infection (42). These three rickettsial species phylogenetically belong to the spotted fever group but are not agents of disease. However, these rickettsiae, by virtue of their abundance, may have a critical role in pathogen-vector interactions.

The presence of known pathogens as well as probable pathogens and symbionts in ticks requires a polymicrobial approach to the clinical aspects of tick-borne infections. Concurrent polymicrobial infections in humans can have a synergistic effect and result in a more severe course of illness. In addition, the clinical course of one tick-borne disease could be influenced simply by exposure to another microbe. For example, A. phagocytophilum and *Ehrlichia* spp. target cells of the innate immune system, and it is possible that even a self-limited transient exposure may influence development of diseases caused by the other pathogens. Excellent reviews have considered the pathogenesis of these organisms (43–46). Of equal importance as the role of polymicrobial infections in clinical disease, the combination of pathogens and symbionts no doubt constitutes a species-specific tick microbiome. There is evidence for detrimental, beneficial, or neutral effects among the interactions of the prokaryotes with each other and with the host tick. These effects can influence access to nutrients, which in turn influence overall fitness of the organisms. For these reasons, and to verify the changing conditions of tick-borne diseases, we performed a polymicrobial assessment of the three species of ticks associated with human disease, including one that is a recent invader. Our results reveal a complex pattern of tick infections with new and emergent pathogens on a background of rapidly shifting tick populations that justify a polymicrobial approach to the study of tick-borne diseases.

## RESULTS

**FIG 1:**
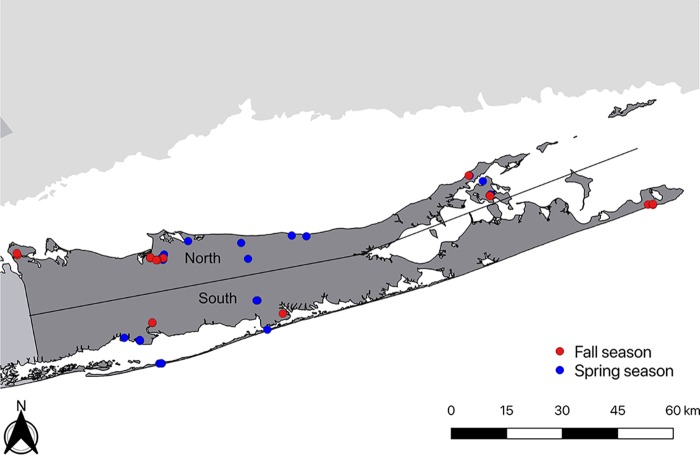
Map of Suffolk County, New York, showing locations of tick collection in the northern and southern regions.

Ticks were collected in the spring and fall of 2018 from multiple locations throughout Suffolk, a suburban county that occupies the central and eastern part of Long Island, NY (https://gisportal.suffolkcountyny.gov/gis/home/). All locations were clustered into northern sites and southern sites (Fig. 1). In the spring, we collected both adults and nymphs of *A. americanum* (676) and I. scapularis (198) and adults of *D. variabilis* (296). Fall collections were comprised of 480 I. scapularis adults. In total, we examined 1,633 individual ticks and 17 pools of 170 *A. americanum* nymphs (10 nymphs per pool). I. scapularis ticks were screened for the presence of B. burgdorferi, B. miyamotoi, A. phagocytophilum, B. microti, POWV, and *Rickettsia* spp. We did not screen I. scapularis ticks for other ehrlichial agents, because they were not detected in a previous study in our area and their geographic range appears to be limited to the Upper Midwest (39). *A. americanum* ticks were screened for *B. lonestari*, *Ehrlichia* spp., and *Rickettsia* spp., as well as for the five agents tested in I. scapularis. *D. variabilis* ticks were screened for *Rickettsia* spp. only.

### Pathogen burden of I. scapularis.

**FIG 2:**
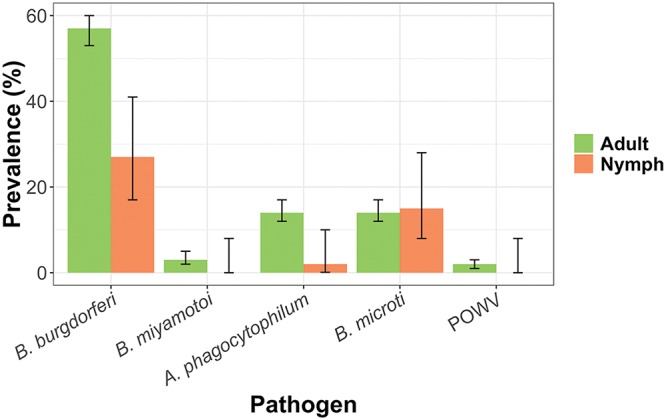
Prevalence of five pathogens in I. scapularis adults and nymphs.

In the northeastern United States, I. scapularis is implicated in the transmission of five agents, including B. burgdorferi, A. phagocytophilum, B. microti, B. miyamotoi, and POWV. All five agents and *R. buchneri* were detected in our study. A total of 430 of 678 (63%) I. scapularis ticks were positive for at least one human pathogen. B. burgdorferi was the most prevalent pathogen in both adults and nymphs (57% and 27%, respectively), followed by B. microti (14% and 15%, respectively) and A. phagocytophilum (14% and 2%, respectively). B. miyamotoi and POWV were detected only in adult ticks and at lower prevalences (3% and 2%, respectively) (Table 1; Fig. 2). We performed additional assays to characterize the strains of A. phagocytophilum and POWV. There are two strains of A. phagocytophilum that differ by 2 bp from their 16S rRNA sequences. One variant (AP variant 1–nonpathogenic) does not infect humans and is carried by deer (47, 48). The HA strain that is implicated in human disease was present in 67 out of 90 (75%) A. phagocytophilum-positive ticks; 19 were found to be of the nonpathogenic strain (21%), and 4 could not be identified further. All POWV strains were part of lineage II, which is associated exclusively with transmission by I. scapularis. In addition to these five agents, we found that of the 187 ticks tested, 87% were positive for *Rickettsia*. Of sequences of PCR products from a 20% subset of the *Rickettsia*-positive ticks, all were identified as *R. buchneri*, an ovarian symbiont. Overall, there were no differences in regional infection rates, with the exceptions of B. microti (*P* = 0.02) and POWV (*P* = 0.01), which were more prevalent in northern Suffolk County (Table 1; Fig. 2). There were no statistically significant differences in infection rates between spring and fall collections in all the pathogens tested for I. scapularis (Table 2). With the exceptions of *R. buchneri* in I. scapularis ticks and *R. amblyommatis* in lone star ticks, adult ticks had significantly higher infection rates with B. burgdorferi and A. phagocytophilum than nymphs (*P* = 0.00003 and *P* = 0.004, respectively) (Tables 1, 2, and 5; Fig. 2). This is a very well-known feature of tick-borne diseases.

**TABLE 1:** Prevalence of five pathogens and *R. buchneri* in I. scapularis ticks by season^*a*^

Agent^*b*^	No. of positive ticks (% [95% CI])		
	Spring (*n* = 198 [140 A/58 N])	Fall (*n* = 480)	Total (*n* = 678 [620 A/58 N])
Borrelia burgdorferi			
A	85 (61 [52–69])	266 (55 [51–60])	351 (57 [53–60])
N	16 (27 [17–41])		16 (27 [17–41])
Borrelia miyamotoi			
A	4 (3 [1–8])	16 (3 [2–5])	20 (3 [2–5])
N	0 (0 [0–8])		0 (0 [0–8])
Anaplasma phagocytophilum			
A	19 (14 [9–21])	70 (15 [12–18])	89 (14 [12–17])
N	1 (2 [0.1–10])		1 (2 [0.1–10])
Babesia microti			
A	27 (19 [13–27])	61 (13 [10–16])	88 (14 [12–17])
N	9 (15 [8–28])		9 (15 [8–28])
POWV			
A	5 (3 [1–8])	6 (1 [0.5–3])	11 (2 [1–3])
N	0 (0 [0–8])		0 (0 [0–8])
Rickettsia buchneri^*c*^			
A	26 (81 [63–92])	64 (89 [79–95])	90 (86 [78–92])
N	21 (91 [70–98])		21 (91 [70–98])

a Total prevalences for both spring and fall seasons are included (excluding *R. buchneri*). Values are means and 95% confidence intervals (CI).

b A, adults; N, nymphs.

c Thirty-two adults and 23 nymphs were tested for the spring season; 72 adult ticks were tested for the fall season.

**TABLE 2:** Prevalence of five pathogens and *R. buchneri* in I. scapularis ticks by geographical region of Suffolk County^*a*^

Agent^*b*^	No. of positive ticks (% [95% CI])			
	Spring		Fall	
	North (*n* = 125 [80 A/45 N])	South (*n* = 73 [60 A/13 N])	North (*n* = 240)	South (*n* = 240)
Borrelia burgdorferi				
A	58 (72 [61–82])	27 (45 [32–58])	139 (58 [51–64])	127 (53 [46–59])
N	12 (27 [15–42])	4 (31 [10–61])		
Borrelia miyamotoi				
A	3 (4 [1–11])	1 (2 [0.1–10])	9 (4 [2–7])	7 (3 [1–6])
N	0 (0 [0–10])	0 (0 [0–28])		
Anaplasma phagocytophilum				
A	11 (14 [7–24])	8 (13 [6–25])	35 (15 [10–20])	35 (15 [10–20])
N	1 (2 [0.1–13])	0 (0 [0–28])		
Babesia microti				
A	19 (24 [15–35])	8 (13 [6–25])	35 (15 [10–20])	26 (11 [7–16])
N	9 (20 [10–35])	0 (0 [0–28])		
POWV				
A	5 (6 [2–15])	0 (0 [0–7])	5 (2 [1–5])	1 (0.4 [0–3])
N	0 (0 [0–10])	0 (0 [0–28])		
Rickettsia buchneri^*c*^				
A	18 (78 [56–92])	8 (89 [51–99])	29 (91 [74–97])	35 (87 [72–95])
N	21 (91 [70–98])			

a Values are means and 95% confidence intervals (CI).

b A, adults; N, nymphs.

c Thirty-two adults and 23 nymphs were tested for the spring season; 72 adult ticks were tested for the fall season.

A total of 22% (147 of 678) of I. scapularis ticks were infected with more than one pathogen. Dual infections were detected in 126 (19%) of the ticks. Triple infections were observed in 21 (3%) of the ticks. A single adult tick collected in the fall was coinfected with four pathogens (Table 3). The highest coinfection prevalence was found with B. burgdorferi and B. microti, with 9% of the total ticks analyzed. Ticks coinfected with B. burgdorferi and A. phagocytophilum accounted for 7% of the total ticks. Triple infections with these three pathogens were more prevalent in both seasons than dual infections with B. burgdorferi-B. miyamotoi, B. burgdorferi-POWV, B. miyamotoi-*A. phagocyotophilum,* and B. miyamotoi-B. microti (Table 3).

**TABLE 3:** Polymicrobial infections detected in I. scapularis ticks

Polymicrobial infections	No. (%) of coinfected I. scapularis ticks		
	Spring (*n* = 198)	Fall (*n* = 480)	Total (*n* = 678)
Two pathogens			
B. burgdorferi-B. miyamotoi	2 (1)	6 (1)	8 (1)
B. burgdorferi-A. phagocytophilum	11 (6)	39 (8)	50 (7)
B. burgdorferi-B. microti	20 (10)	41 (8)	61 (9)
B. burgdorferi-POWV	2 (1)	1 (0.2)	3 (0.4)
B. miyamotoi-A. phagocytophilum		3 (0.6)	3 (0.4)
B. miyamotoi-B. microti		1 (0.2)	1 (0.1)
Three pathogens			
B. burgdorferi-B. miyamotoi-A. phagocytophilum		2 (0.4)	2 (0.3)
B. burgdorferi-A. phagocytophilum-B. microti	5 (2)	2 (0.4)	7 (1)
B. burgdorferi-B. microti-POWV	2 (1)	1 (0.2)	3 (0.4)
A. phagocytophilum-B. microti-POWV		1 (0.2)	1 (0.1)
Four pathogens			
B. burgdorferi-B. miyamotoi-A. phagocytophilum-B. microti		1 (0.2)	1 (0.1)

Coinfection of A. phagocytophilum and B. microti with B. burgdorferi in host-seeking I. scapularis ticks has been reported in several regions of the United States (Table 4). The 54%, 14%, and 13% prevalences *of* B. burgdorferi, B. microti, and A. phagocytophilum, respectively, in this study are consistent with the prevalence rates found in New York State (39, 49, 50) but are higher than those of other regions in the Northeast (Table 4). The insularity of Long Island could explain and be a determinant of the high global prevalence rate of infected I. scapularis ticks found in this study, since rodent population densities inhabiting this region could be affected by the island syndrome (51) that favors increases in rodent populations, which, in turn, could lead to greater opportunities for juvenile stages of I. scapularis to find a rodent host and therefore get infected by one or more pathogens.

**TABLE 4:** Prevalence of the most common single infections and pathogen coinfections reported in questing I. scapularis ticks in the United States from 2003 to 2017

Location	Yr(s)	No. of ticks	Stage^*a*^	Single infections (%)^*b*^			Double infections (%)		Triple infections (%) of Bb-Apc-Bm	Reference
				Bb	Ap^*c*^	Bm	Bb-Ap^*c*^	Bb-Bm		
NJ	2003–2004	147	A	50	6		3			115
NY	2003–2006	3,300	N	14	6	3	0.5	1		116
		7,914	A	46	12	2	6	1	0.4	116
ME	2003	100	A	58	16	7	9	11^*d*^		117
IN	2004	100	A	72	5		4	4^*d*^		117
PA	2005	94	A	52	1		1	1^*d*^		117
WI	2006	100	A	35	14		8	4^*d*^		117
MI	2006	119	A	50	4		2			118
NJ	2004–2007	478	N	10		4	3			119
		610	A	45		8	6			
IA	2007–2009	156	N	17	29		6			120
NY	2008	132	A	62	22	26	16	22	7	49
CT	2008	154	A	65	17	16	16	12	3	49
WI	2009–2013	748	N	29	5		3			121
NY	2011	323^*e*^	N	67	34	19				50
		922^*e*^	A	60	23	4				
		466	A	55	18	3	10	1	1	
NY	2011–2012	4,368	N	19	5	6	2	7	1	122
CT	2011–2012	514	N	13	3	6	1	2		123
MD	2011–2012	124	N	19	1					123
NY	2011–2012	207	N	23	5	11		6		123
PA	2013	1,363	A	47	3	3	1	2		124
MD	2014–2015	168	N	21	1					125
NY	2014–2015	299	N	17	9	3	3	4		125
PA	2014–2015	114	N	22	3		2			125
VA	2014–2015	472	N	12	1		1			125
DC	2014–2015	253	N	23	4					125
ME	2015	154	N	18	3	4		3		125
MN	2015	1,240	N	25	6	5	2	2	1	126
WI	2015	112	A	41	11	9	1	1	1	127
PA	2015–2017	1,721	N	25	1	3	1	1		128
NY	2016–2017	197	A	56	11	8	4	0.5	2	39

a N, nymphs; A, adults.

b Bb, Borrelia burgdorferi; Ap, Anaplasma phagocytophilum; Bm, Babesia microti.

c Ap human variant.

d Coinfection of B. burgdorferi and B. microti plus Babesia odocoilei combined.

e Tested in pools of single individuals to a maximum of 10.

### Pathogen burden of *A. americanum*.

The aggressive species *A. americanum* is implicated in the transmission of the causative agents of ehrlichiosis (Table 5). We detected *Ehrlichia* spp. in 23 (7%) of adult *A. americanum* ticks. *E. ewingii* was the predominant species in our ticks (12 ticks; 4%), followed by *E. chaffeensis* (6 ticks; 2%), and an Ehrlichia ruminantium-like species (5 ticks; 1%). We also detected *Ehrlichia* in 4 out 354 individual *A. americanum* nymphs. Two nymphs were positive for *E. ewingii* and 2 for *E. chaffeensis*. All pools of *A. americanum* nymphs were negative for *Ehrlichia*.

**TABLE 5:** Agents detected in *A. americanum* and *D. variabilis* ticks^*a*^

Tick species and agent^*b*^	No. of positive ticks (% [95% CI])		
	Spring		Total
	North	South	
*Amblyomma americanum*	*n* = 352 (128 A/224 N)	*n* = 324 (194 A/130 N)	*n* = 676 (322 A/354 N)
*Borrelia lonestari*^*c*^			
A	1 (1 [0.05–6])	5 (3 [1–8])	6 (2 [1–5])
N	1 (0.5 [0.2–3])	1 (1 [0.5–6])	2 (0.6 [0.1–3])
Ehrlichia chaffeensis			
A	3 (2 [0.6–7])	3 (1 [0.4–5])	6 (2 [0.7–4])
N	1 (0.4 [0.2–3])	1 (0.8 [0.4–5])	2 (0.6 [0.1–2])
Ehrlichia ewingii			
A	8 (6 [3–12])	4 (2 [0.7–5])	12 (4 [2–6])
N	2 (1 [0.1–3])	0 (0 [0–3])	2 (0.6 [0.1–2])
*E. ruminantium*-like species			
A	2 (2 [0.3–6])	3 (1 [0.4–5])	5 (1 [0.6–4])
N	0 (0 [0–2])	0 (0 [0–3])	0 (0 [0–1])
Total *Ehrlichia* spp.			
A	13 (10 [6–17])	10 (5 [3–9])	23 (7 [5–11])
N	3 (1 [0.3–4])	1 (0.8 [0.4–5])	4 (1 [0.4–3])
Rickettsia amblyommatis			
A	79 (62 [53–70])	119 (61 [54–68])	198 (61 [56–67])
N	111 (49 [43–56])	85 (65 [56–73])	196 (55 [50–60])
Dermacentor variabilis	*n* = 124	*n* = 172	*n* = 296
Rickettsia montanensis	6 (5 [2–11])	2 (1 [0.2–5])	8 (3 [1–5])

a Values are means and 95% confidence intervals (CIs).

b A, adult; N, nymph.

c A total of 548 ticks were tested from the northern (102 adults, 210 nymphs) and southern (140 adults, 96 nymphs) regions of Suffolk County.

*B. lonestari* was present in 8 ticks (1%). We did not detect B. burgdorferi in *A. americanum*, reasserting that this tick species does not play a role in its transmission. Early reports of the Lyme disease agent in this tick may have been mistaken with *B. lonestari* (52). None of the *A. americanum* ticks had detectable B. microti, B. miyamotoi, A. phagocytophilum, or POWV.

We detected a single *Rickettsia* species in *A. americanum*, identified as *R. amblyommatis*. More than half of *A. americanum* (58%) ticks were positive for *R. amblyommatis*.

### Impact of tick-borne blood infections.

Three pathogens detected in this study infect blood cells. A. phagocytophilum infects neutrophils, B. microti infects erythrocytes, and the *Ehrlichia spp.* infect monocytes. All three pathogens can also be transmitted by transfusion of blood and blood products (53–55).

**FIG 3:**
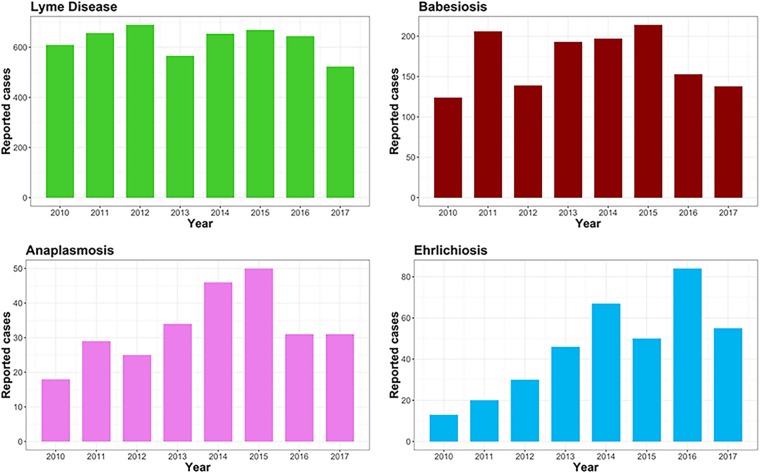
Number of cases of anaplasmosis, babesiosis, ehrlichiosis, and Lyme disease from 2010 to 2017 in Suffolk County, New York. Numbers were derived from https://www.health.ny.gov/statistics/diseases/communicable/.

The ratio of Lyme disease cases to babesiosis cases has been approximately 4 to 1 for the last 8 years, higher than in other areas where I. scapularis is endemic (Fig. 3; Table 4). This ratio also holds for the ratio of B. burgdorferi to B. microti in ticks in this study, i.e., 3.8 to 1 (Tables 1 and 2). Conversely, during the same 8-year period, the ratio of Lyme disease cases to cases of anaplasmosis is approximately 15 to 1, but our data showed a 4-to-1 ratio of B. burgdorferi to A. phagocytophilum. This disparity between the reported cases of anaplasmosis and the tick infection rates is not readily explainable but could be due to the role that the nonpathogenic strain might have in exposure. Alternatively, a larger number of anaplasmosis cases may not be severe enough to warrant medical attention.

There are increasing numbers of reported cases of ehrlichiosis in Suffolk County (Fig. 3). Although the infection rate of *Ehrlichia* spp. in *A. americanum* nymphs and adults was less than 5% (Table 5), the growing populations of these ticks as well as their aggressive nature create additional anxiety over this emerging infection (Fig. 3).

The three tick-borne blood infections have several features in common. The frequency of reported cases of all three is highest in the elderly (anaplasmosis susceptibility appears to be less bound by age) and in males. Importantly, people at risk for clinical infection and for increased disease severity have compromised immune systems due to a number of causes, which can include cancer therapy, immunosuppressive drugs to treat autoimmune disorders, splenectomy, preexisting liver disease, and endogenous disorders of the immune system, among others. In communities with both high tick infestations and the presence of a sizable elderly population, the features of the three blood infections are important contributors to their high morbidity and mortality (https://www.cdc.gov/anaplasmosis/stats/index.html; https://www.cdc.gov/parasites/babesiosis/disease.html; https://www.cdc.gov/ehrlichiosis/stats/index.html).

### The genus *Rickettsia* constitutes the largest biomass in the three tick species.

*R. montanensis* (2.7%) was the only agent detected in *D. variabilis* (Table 5). The cases of RMSF have decreased markedly in Suffolk County since the 1970s, with the numbers of cases ranging from 2 to 14 per year in the last decade (https://www.health.ny.gov/statistics/diseases/communicable/). *R. amblyommatis* was present in more than half of the *A. americanum* ticks sampled in this study (Table 5). *R. buchneri* is an ovarian endosymbiont with an overwhelming presence in I. scapularis (Table 1; Fig. 2) and has not been associated with human disease. However, its presence could be a factor in the associations with pathogens found simultaneously in this tick.

## DISCUSSION

The polymicrobial infection approach to tick-borne diseases is rooted in the discovery of new or emergent pathogens, such as the ones detected in this study, changes in or discovery of new pathogen genotypes, such as is the case for POWV and A. phagocytophilum, and expansion of tick ranges as well as of their pathogens, as represented by *A. americanum.* The changes in tick populations and their pathogens may be the result of climate change, with unpredictable short- or long-term consequences. However, factors other than climate change, such as anthropogenic changes and host availability, might have great impact.

### Ixodes scapularis microbiome.

I. scapularis is a public health threat (56). B. burgdorferi infects more than half of the adult I. scapularis ticks tested, and this accounts for the high number of Lyme disease cases in our area (Fig. 3). Of note is that nearly one-quarter of the I. scapularis ticks tested had polymicrobial infections, and this justifies the modification of the clinical approach to tick-borne diseases to cover all infection possibilities. At a more fundamental level, the polymicrobial infections open new possibilities for research into the microbial relationships with and within the tick.

Babesia microti causes clinical disease in elderly, immunosuppressed, and splenectomized patients (57, 58) and also in blood transfusion recipients (59). Asymptomatic babesiosis exists in individuals that do not have the above risk factors, representing a group of seropositives, and these represent a major risk as blood transfusion donors. Genomic tools have added new perspectives on the expansion of B. microti (60). There are Northeast and Midwest lineages segregating into local populations of the parasite (61, 62), suggesting dynamic adaptation changes promoting expansion.

Anaplasmosis is variable in terms of severity. The two variants of A. phagocytophilum also have ecological implications, in that the AP-HA variant is acquired from mice, whereas the reservoir of the nonpathogenic variant is deer. That these variants were detected in our study is representative of the advances at the molecular biology level that have a bearing on epidemiology.

POWV is the causative agent of a life-threatening encephalitis (63). There have not been any reported cases of POWV in our area, but its low-level presence (lineage II) in I. scapularis could result in transmission to humans. Lineage II virus circulates in an I. scapularis–white-footed mouse cycle, and it is precisely in this cycle that this virus can become a part of the polymicrobial group of pathogens (63–69).

### *Amblyomma americanum* expansion.

Since the first confirmed records (22), and thereafter (23, 70), the presence of *A. americanum* has increased markedly, and it is likely the most abundant tick species in our environment. However, the lack of systematic tick surveillance in the intervening years since the first records were made makes this difficult to confirm. The expansion of *A. americanum* into the North Atlantic states has been mapped and documented (20, 21, 52, 71). Sonenshine connected the northward expansion of *A. americanum* to climate change (72). This expansion has occurred in a short time. An increase of nearly 60% in *A. americanum* numbers has been reported in Connecticut in the last 20 years (73), as well as in New Jersey (74). Studies in our laboratory showed that genetic changes occur in the tick population during range expansion (75).

There are serious consequences to the range expansion of *A. americanum*. Notably, even in the absence of transmitted pathogens, this species is an aggressive and tenacious tick. All three stages bite humans, and the larvae, in particular, can infest in large numbers, leading to an uncomfortable dermatitis. There are some subtle effects as well. Large populations of *A. americanum* in a community can lead to an exaggerated perception of the risk of Lyme disease, as most people cannot differentiate between I. scapularis and lone star ticks (76).

The cases of ehrlichiosis have increased steadily in our area in recent years as a function of the increases in the populations of *A. americanum* (Fig. 3). Our results indicate that there are three *Ehrlichia* species in Suffolk County. *E. ewingii* is the most frequent *Ehrlichia* species present in our ticks, and therefore, it could be the main causative agent of human ehrlichiosis in this area (49) (Table 5). *E. chaffeensis* is clinically indistinguishable from *E. ewingii*, and species identification is not possible by serology, so it may be difficult to determine the infecting species (77). An *E. ruminantium-*like species is present in *A. americanum* in our area, although its disease-causing potential is unclear (78). Transfusion-acquired *E. ewingii* has been documented (79), making this organism a serious threat to recipients who are at greater risk of a severe infection (80), and infection with *E. ewingii* has been documented in patients that were not immunosuppressed. Pediatric ehrlichiosis with an increased case fatality rate has been documented as well (81).

*B. lonestari* has been detected at low levels, but STARI has not been documented in our area as it has elsewhere in areas where *A. americanum* is abundant (82–84). *B. lonestari* has been linked as the causative agent of STARI from a single patient with erythema migrans (85). However, to date, this has been the only case reported.

### Rickettsial biome.

The rickettsiae constitute the largest prokaryotic biomass in two of the three species of ticks detected here. The sheer abundance of these rickettsiae can be expected to have a major role in the vector-pathogen relationship and to influence the pathogen-patient relationship after transmission.

There was overwhelming infection of *R. amblyommatis* (40) in our *A. americanum* populations. This rickettsia may be more than a symbiont, as cases of rickettsiosis may have been caused by *R. amblyommatis* (86). RMSF-like illness may be associated with the expanding range of *A. americanum* (21). This is further supported by the lack of detection of *R. rickettsii* in tick surveillance studies, including the work presented here. However, that there is a high prevalence of *R. amblyommatis* does not necessarily support its role in human disease. Whether a symbiont, a pathogen under certain conditions, or an outright pathogen, *R. amblyommatis* has introduced a confounding factor in the diagnoses of febrile tick-borne infections, as positive serologies may be misinterpreted.

The rickettsiae present in the ovaries of I. scapularis belong to the spotted fever group and are closely related to *R. montanensis* (36, 87, 88). The most abundant prokaryotic DNA sequences found in I. scapularis were from *R. buchneri* (37–39). *R. buchneri* underwent the introduction of transposons with genomic deletions and mutations that resulted in the loss of pathogenicity (89). *R. buchneri* contains multiple mobile genetic elements that endow this species with a plastic and repetitive genome that is thought to account for its symbiotic lifestyle (90). Its overwhelming presence in this study suggests that this organism plays an undisputed role in the homeostasis of this tick.

*R. montanensis* was the only agent detected in *D. variabilis* ticks, with no detection of *R. rickettsii*. This tick was associated with an outbreak of RMSF in our area in the late 1970s (91). Given our findings in this study, we support the suggestion that other rickettsial species could confound a diagnosis of RMSF (92, 93), although the prevalence of this symbiotic organism in *D. variabilis* seems to have remained stable for many years (92).

Polymicrobial infections may be synergistic in enhancing the severity of human illnesses, and more-deleterious interactions could be discovered if there was greater emphasis on a pluralistic approach to tick-borne diseases. There is an extensive literature on the topic of coinfections, and we cite the most recent for American patients (94) and for Europe (95). However, despite the high levels of tick coinfections, there is at least one therapeutic feature that could mitigate the impact of polymicrobial infections in patients. Two of the most common coinfecting organisms, B. burgdorferi and A. phagocytophilum, are responsive to doxycycline, so both could be treated simultaneously. Polymicrobial infections can lead to treatment problems as well. The possibility of side effects has resulted in doxycycline not being prescribed to young children, an overrepresented group in Lyme disease. B. microti requires antiparasitic drugs that do not work against B. burgdorferi and A. phagocytophilum. Likewise, beta-lactam antibiotics can be active against B. burgdorferi but not A. phagocytophilum, and it is in these cases where the complexity of polymicrobial infections requires further treatment evaluation.

Nonetheless, the relationships of pathogens and symbionts in the tick have biological significance but appear to be complex. Pathogen burden could be important in maintaining the balance of organisms within the tick, where deviations may alter the mechanics of transmission. On one hand, there is evidence for the possibility of limited interactions among the several coinfecting pathogens in I. scapularis. These pathogens occupy different anatomical niches in the tick, as some are extracellular in the lumen of the midgut (*Borrelia*) and others are obligate intracellular organisms (*Anaplasma*, *Babesia*, and POWV), so it is possible that direct contact among them may be limited. There is a hierarchy of transmission of pathogens in relation to the duration of blood feeding. POWV can be transmitted within 15 min of tick attachment, and both A. phagocytophilum and B. miyamotoi can be transmitted within the first 24 h of attachment. Transmission of B. burgdorferi increases with the length of attachment (96–98), and B. microti needs to change its morphology while in the salivary glands of the tick, requiring a longer period of time for transmission. This pattern of staggered transmission may further segregate the pathogens within the vector and actually favor acquisition of the organisms that are transmitted quickly, as people may remove ticks in time to abort infections with the slower pathogens.

Notwithstanding, there is increasing evidence for far-reaching interactions among symbionts and pathogens with each other and, collectively, with the ticks. In some instances, the interaction can be to the benefit of the pathogen. A. phagocytophilum induces the production of the antifreeze glycoprotein (IAFGP), which in turn makes infection easier (99). Relationships between the symbiont-pathogen and the tick may result in a neutral status quo; for example, the vector competence of *A. americanum* for *R. rickettsii* was not significantly affected by *R. amblyommatis* (100). In other instances, tick responses can be harmful to pathogens. Hemocytes of I. scapularis ingest B. burgdorferi (101), and this tick regulates infection with A. phagocytophilum through the production of antimicrobial peptides, and actin phosphorylation for survival, as well as proteases to increase vector fitness (102–104). *R. montanensis* (an organism of unproven pathogen status) induces ticks to produce defensins (105) and protease inhibitors that limit colonization by this *Rickettsia* species (106). *Anaplasma*, *Borrelia*, *Ehrlichia,* and *Rickettsia* spp. do not have interbacterial effector and immunity genes whose products regulate interactions among bacteria utilizing the same niche (38). The lack of these genes can lead to a system of shared tolerance for each other that would be clearly to their advantage for survival in the vector. It appears that we have only begun to appreciate the interactions of the microbes with each other and with the tick. The best approach to study the interactions among the prokaryotes in the tick is to consider the polymicrobial nature of these fascinating biological systems (107–109).

## MATERIALS AND METHODS

### Tick collection and study areas.

Two active tick-borne pathogen surveillance programs were conducted during the spring season peak (from May to July) and the fall season peak (from October to November) throughout Suffolk County, New York, in 2018. Questing ticks were collected from vegetation along trails by flagging a 1-m^2^ cotton flannel fabric attached on both ends to a wooden pole between 10:00 and 14:00 h during sunny days. The area of study was divided into two geographical regions to compare potential differences in infection rates between the northern and the southern regions of the county (Fig. 1). Ticks were collected for a minimum of 60 min per site in order to collect a representative number of each species to estimate the prevalence of pathogens in each geographical region.

All collected ticks were identified morphologically to species, life stage, and sex by use of a dissecting microscope and the appropriate taxonomic keys and stored at –80°C until further processing (110–112).

### TNA extraction from ticks.

Total nucleic acids (TNA) were extracted using the NucliSENS easyMAG platform (bioMérieux, Durham, NC). Ticks were grouped by location and day of collection. To remove environmental contaminants, pools of 10 ticks were washed with 3% hydrogen peroxide, followed by three washes with 1 ml of 1× phosphate-buffered saline (PBS). The ticks were then placed individually in a sterile 1.5-ml centrifuge tube and homogenized using a 21-gauge, 1.5-in needle in 50 μl of 1× PBS. The entire volume was then added to NucliSENS easyMAG lysis buffer (bioMérieux, Durham, NC). TNA were extracted according to the manufacturer’s protocol, eluted in 40 μl, and stored at –80°C.

### Pathogen detection by real-time PCR.

We utilized a pathogen detection strategy in which we tested each tick TNA by quantitative PCR (qPCR) using two approaches, consisting of either a single-agent qPCR using DNA as a template or a multiplex one-step reverse transcription PCR (RT-qPCR), using both cDNA and DNA as a template (113).

All I. scapularis and *A. americanum* samples were screened for the presence of B. burgdorferi, B. miyamotoi*/B. lonestari*, B. microti, A. phagocytophilum, and POWV with the multiplex RT-qPCR (Table 6). Our rationale for testing *A. americanum* was to evaluate the role of this tick as a potential vector of these five agents. For positive controls of the multiplex assay, we employed quantified plasmid standards at 10, 100, and 1,000 copies (113). All remaining tests consisted of single-agent qPCR assays. For detection of *Rickettsia*, we developed a qPCR assay designed to detect a fragment within the *ompB* gene of the most common spotted fever group *Rickettsia* species. This assay was used to screen all TNA from *A. americanum* and *D. variabilis* ticks and from 127 I. scapularis ticks. For positive controls, we used TNA from an I. scapularis adult previously shown to be infected with *R. buchneri*. To detect *Ehrlichia* spp., we employed an *Ehrlichia*-specific assay targeting a portion of the 16S rRNA gene. This assay was used to test all *A. americanum* ticks (Table 6). For positive controls, we used DNA from a lysate of *E. chaffeensis*-infected DH82 cells.

**TABLE 6:** Primer and probe sequences for RT-PCR^*a*^

Pathogen	Gene target	Primer pair	Probe	5′ dye	3′ quencher
Borrelia burgdorferi	*ospA*	Fwd: CCTTCAAGTACTCCAGATCCATTG	CAACAGTAGACAAGCTTGA	6-FAM	MGB
		Rev: AACAAAGACGGCAAGTACGATC			
Borrelia miyamotoi*/B. lonestari*	*flaB*	Fwd: AGCACAAGCTTCATGGACATTGA	TGTGGGTGCAAATCAGGATGAAGCA	HEX	BHQ-1
		Rev: GAGCTGCTTGAGCACCTTCTC			
Babesia microti	*cox1*	Fwd: CATCATGCCAGGCCTGTTTG	TACTACCCATACTGGTCGGTGCTCC	Quasar 705	BHQ-2
		Rev: GAAGAAACCACAAGAGCAAATGC			
Anaplasma phagocytophilum	16S rRNA	Fwd: GGCATGTAGGCGGTTCGGT	GCCAGGGCTTAACCCTGGAGCT	Cy5	BHQ-2
		Rev: CACTAGGAATTCCGCTATCCTCTCC			
Powassan virus	3′UTR	Fwd: GTGATGTGGCAGCGCACC	CCTACTGCGGCAGCACACACAGTG	Texas Red	BHQ-2
		Rev: CTGCGTCGGGAGCGACCA			
*Ehrlichia* spp.	16S rRNA	Fwd: CGTAAAGGGCACGTAGGTGGACTA	TCGAAAGAGGATAGCGGA	VIC	MGB
		Rev: CACCTCAGTGTCAGTATCGAACCA			
*Rickettsia* spp.	*ompB*	Fwd: AACAAGCTGCTGGGCACCATAT	AGAGAATGAGAAACCGTTAACGT	FAM	MGB
		Rev: CGGTGCTGCTATCGGTATCACT			

a UTR, untranslated region; 6FAM, 6-carboxyfluorescein; HEX, 6-carboxy-2,4,4,5,7,7-hexachlorofluorescein; BHQ, black hole quencher.

All qPCRs were performed on a Bio-Rad C1000 Touch system with a CFX96 optical module (Bio-Rad, Hercules, CA) using the RNA UltraSense one-step quantitative RT-PCR system (Invitrogen, Carlsbad, CA). The final reaction mixture contained 5 μl of template and 20 μl of master mixture. The master mixture contained 0.2 μM each forward primer, 0.3 μM each reverse primer, 0.1 μM each probe, 1.25 μl of RNA UltraSense enzyme mix, and 5 μl RNA UltraSense 5× reaction mix. The reverse transcription step was performed at 55°C for 15 min, followed by incubation at 95°C for 10 min. The RT step was omitted for the *Ehrlichia* and *Rickettsia* qPCR assays. The PCR consisted of 40 cycles at 95°C for 15 s and 60°C for 30 s.

### Qualitative PCR.

We developed qualitative PCR assays to further characterize the species detected by qPCR. For *Rickettsia*, we employed an assay that amplified 380 nucleotides (nt) within *ompB*. For *Anaplasma*, we designed an assay within *gltA* to differentiate between the pathogenic agent (AP-HA) and nonpathogenic variants (AP-variant 1). For *Ehrlichia*, we first employed an assay targeting the 16S rRNA gene. Any samples found to be positive for the *E. ruminantium* variant were tested with a nested PCR targeting the *gltA* gene.

For characterization of B. burgdorferi, B. miyamotoi, *B. lonestari*, and POWV, we used our previously established assays (49, 114).

All qualitative PCRs were performed using AmpliTaq Gold 360 master mix (Applied Biosystems, Foster City, CA) in a 25 μl-reaction mixture with 2 μl of template, 0.2 μM forward primer, and 0.3 μM reverse primer and 12.5 μl of the polymerase. Primers and reaction conditions used for each pathogen are described in Table 7. A nontemplate control was included in each assay. PCR products were resolved through 2% agarose gel in Tris-borate-EDTA (TBE) buffer and sequenced by dideoxy sequencing. All sequences were analyzed using Geneious 10.0.9 software.

**TABLE 7:** Primer sequences and PCR conditions for qualitative assay

Pathogen	Gene target	Primer pair	Product length (nt)	PCR conditions
Borrelia burgdorferi	*ospA*	Fwd: GCGTTTCAGTAGATTTGCCT	676	95°C for 10 min; 40 cycles of 95°C for 30 s, 57°C for 30 s, and 72°C for 60 s
		Rev: TTGGTGCCATTTGAGTCGTA		
Borrelia miyamotoi*/* *B. lonestari*	*flaB*	Fwd: GGGATTATMAATCATAATACRTCAGC	967 (B. miyamotoi)/ 949 (*B. lonestari*)	95°C for 10 min; 40 cycles of 95°C for 30 s, 57°C for 30 s, and 72°C for 70 s
		Rev: TTGCTTGTGCAATCATAGCCATTGC		
Babesia microti	18S rRNA	Fwd: GGGACTTTGCGTTCATAAAACGC	171	95°C for 10 min; 40 cycles of 95°C for 30 s, 60°C for 30 s, and 72°C for 45 s
		Rev: GCAATAATCTATCCCCATCACGAT		
Anaplasma phagocytophilum	*gltA*	Primary reaction	668	95°C for 10 min; 40 cycles of 95°C for 30 s, 62°C for 30 s, and 72°C for 60 s (same conditions for primary and secondary reactions)
		Fwd: ACCGGAACCCCCATAGCTCT		
		Rev: GCAAGTCGCATTGATCCGCT		
		Secondary reaction	589	
		Fwd: TCGACATTTGGGTACAACTTGCG		
		Rev: CAATTGCGAAAGTACCCGGCA		
Powassan virus	Envelope (E) glycoprotein	Fwd: GGCAACTGCATCTCTATRAATCC	395	95°C for 10 min; 40 cycles of 95°C for 30 s, 58°C for 30 s, and 72°C for 45 s
		Rev: CCTCATGCAGTGAAAATGGATATCTT		
*Ehrlichia* spp.	16S rRNA	Fwd: ATGCGTAGGAATCTACCTAGTAGTA	460	
		Rev: GCCTTGGTATTTCACTTTTAACTTACT		
*Ehrlichia* spp.	*gltA*	Primary reaction	777	95°C for 10 min; 40 cycles of 95°C for 30 s, 56°C (primary)/58°C (secondary) for 30 s, and 72°C for 60 s
		Fwd: CCAGGATTTATGTCTACTGCTGC		
		Rev: GCATACYCTATGACCAAAMCCCAT		
		Secondary reaction	627	
		Fwd: GCGCGGATTACRTTTATTGATGG		
		Rev: ATTGGCHCCACCATGAGCTG		
*Rickettsia* spp.	*ompB*	Fwd: GGTACTGCCGAGTTACGTTTAG	380	95°C for 10 min; 40 cycles of 95°C for 30 s, 57°C for 30 s, and 72°C for 45 s
		Rev: CTCGCATCAACAACRCCTG		

### Statistical analysis.

A Fisher’s exact test was performed for each agent to compare seasonal and geographical infection rates with the five pathogens. A two-tailed *P* value of <0.05 was considered statistically significant. Statistical analyses were performed using the software package R (R-Development Core Team; www.r-project.org). The mean and 95% confidence intervals of pathogen prevalence were calculated using the proportion test in R.
